# Extension of the Kuramoto model to encompass time variability in neuronal synchronization and brain dynamics

**DOI:** 10.1186/1471-2202-12-S1-P313

**Published:** 2011-07-18

**Authors:** Spase Petkoski, Aneta Stefanovska

**Affiliations:** 1 Department of Physics, Lancaster University, Lancaster, LA1 4YB, UK

## 

The Kuramoto model (KM) is extended to incorporate at a basic level one of the most fundamental properties of living systems – their inherent time-variability. In building the model, we encompass earlier generalizations of the KM that included time-varying parameters in a purely physical way [1,2] together with a model introduced to describe changes in neuronal synchronization during anæsthesia [3], as one of the many experimentally confirmed phenomena [4,5] which this model should address. We thus allow for the time-variabilities of both the oscillator natural frequencies and of the inter-oscillator couplings. The latter can be considered as describing in an intuitive way the non-autonomous character of the individual oscillators, each of which is subject to the influence of its neighbors. The couplings have been found to provide a convenient basis for modeling the depth of anæsthesia [3].

Non-autonomous natural frequencies in an ensemble of oscillators, on the other hand, have already been investigated and interpreted as attributable to external forcing [6]. Our numerical simulations have confirmed some interesting, and, at first sight counter-intuitive, dynamics of the model for this case, and have also revealed certain limitations of this approach. Hence, we further examine the other aspects of the frequencies’ time-variability. In addition, we apply the Sakaguchi extension (see [3] and the references therein) of the original KM and investigate its influence on the system’s synchronization. Furthermore, we propose the use of a bounded distribution for the natural frequencies of the oscillators. A truncated Lorentzian distribution appears to be a good choice in that it allows the Kuramoto transition to be solved analytically: the resultant expression for the mean field amplitude matches perfectly the results obtained numerically.

The work to be presented helps to describe time-varying neural synchronization as an inherent phenomenon of brain dynamics. It accounts for the experimental results reported earlier [4] and it extends and complements a previous attempt [3] at explanation.
